# How well do services for young people with long term conditions deliver features proposed to improve transition?

**DOI:** 10.1186/s12913-018-3168-9

**Published:** 2018-05-08

**Authors:** A. Colver, R. Pearse, R. M. Watson, M. Fay, T. Rapley, K. D. Mann, A. Le Couteur, J. R. Parr, H. McConachie, on behalf of the Transition Collaborative Group, Caroline Bennett, Gail Dovey-Pearce, Greg Maniatopoulos, Janet McDonagh, Debbie Reape, Luke Vale, Nichola Chater, Helena Gleeson, Anastasia Bem, Stuart Bennett, Amanda Billson, Stephen Bruce, Tim Cheetham, Diana Howlett, Zilla Huma, Mark Linden, Maria Lohan, Melanie Meek, Jenny Milne, Julie Owens, Fiona Regan, Nandu Thalange

**Affiliations:** 1 Institute of Health and Society, Newcastle University, Newcastle upon Tyne, NE1 4LP UK; 2 Northumbria Healthcare NHS Foundation Trust, North Shields, NE29 8NH UK; 3 Department of Social Work, Education and Community Wellbeing, Northumbria University, Newcastle upon Tyne, NE7 7XA UK; 4 Centre for Neurorehabilitation and Neuropsychiatry, Northumberland Tyne and Wear NHS Foundation Trust, Newcastle upon Tyne, NE6 4QD UK; 5 Institute of Neuroscience, Newcastle University, Newcastle upon Tyne, NE1 4LP UK

**Keywords:** Transition, Long term conditions, Young people, Parent involvement, Key worker, Chronic illness, Disability, Features of services

## Abstract

**Background:**

For young people with long-term conditions, transition from child to adult-oriented health services is a critical period which, if not managed well, may lead to poor outcomes. There are features of transition services which guidance and research suggest improve outcomes. We studied nine such features, calling them ‘proposed beneficial features’*: age-banded clinic; meet adult team before transfer; promotion of health self-efficacy; written transition plan; appropriate parent involvement; key worker; coordinated team; holistic life-skills training; transition manager for clinical team*. We aimed to describe the extent to which service providers offer these nine features, and to compare this with young people’s reported experience of them.

**Methods:**

A longitudinal, mixed methods study followed 374 young people as their care moved from child to adult health services. Participants had type 1 diabetes, cerebral palsy or autism spectrum disorder with additional mental health difficulties. Data are reported from the first two visits, one year apart.

**Results:**

Three hundred four (81.3%) of the young people took part in the second visit (128 with diabetes, 91 with autism, 85 with cerebral palsy). Overall, the nine proposed beneficial features of transition services were poorly provided. Fewer than half of services stated they provided an *age-banded clinic*, *written transition plan*, *transition manager for clinical team*, a *protocol for promotion of health self-efficacy*, or *holistic life-skills training.*

To varying degrees, young people reported that they had not experienced the features which services said they provided. For instance, the agreement for *written transition plan*, *holistic life-skills training* and *key worker*, was 30, 43 and 49% respectively. Agreement was better for *appropriate parent involvement,* a*ge-banded clinic,* p*romotion of health self-efficacy* and *coordinated team* at 77, 77, 80 and 69% respectively. Variation in the meaning of the features as experienced by young people and families was evident from qualitative interviews and observations.

**Conclusions:**

UK services provide only some of the nine proposed beneficial features for supporting healthcare transition of young people with long term conditions.

Observational studies or trials which examine the influence of features of transition services on outcomes should ensure that the experiences of young people and families are captured, and not rely on service specifications.

**Electronic supplementary material:**

The online version of this article (10.1186/s12913-018-3168-9) contains supplementary material, which is available to authorized users.

## Background

‘Transition’ is a wider concept than transfer from child to adult healthcare, and is defined as ‘the purposeful, planned process that addresses the medical, psychosocial, educational and vocational needs of adolescents and young adults with chronic medical and physical conditions’ [1].

Policy documents from professional bodies [2], UK Government [3] and the National Institute for Health and Care Excellence (NICE) [4] indicate that Transition is important, is undertaken poorly in many instances, and can be improved.

Accumulating research evidence [4, 5] suggests that there are features of transition services for young people that may improve outcomes. However, we do not know whether services for young people offer such features, or whether these features are experienced by young people during transition. Recommended features may not be introduced even in services where improving transition processes has been a focus, such as those for young people with type 1 diabetes [6]. Garvey et al. [7] surveyed young adults after transfer to adult diabetes care and found that fewer than 15% had received written transition plans or met the adult provider before transition. A survey of UK child and adolescent mental healthcare found that only 5% of young people experienced ‘optimal transition’ (joint planning, information transfer, continuity of care) to adult mental health services [8, 9].

The Transition Research Programme was funded by the UK National Institute for Health Research to examine the process of transition for young people with long term conditions. The Programme included a longitudinal, mixed methods study which recruited 374 young people and followed them over three years as their clinical care moved from child to adult health services. In developing the protocol, policy documents and published evidence were studied, resulting in the identification of nine service features which appeared to be associated with improved outcomes [10]. The features are applicable to services across health conditions. As evidence about the effectiveness of these service features is incomplete [11], we use the term ‘proposed beneficial features’ (PBFs) to describe them in this paper. The nine PBFs we examined were: *age-banded clinic*; *meet adult team before transfer*; *promotion of health self-efficacy*; *written transition plan*; *appropriate parent involvement; key worker; coordinated team*; *holistic life-skills training; transition manager for clinical team*. We think it is important, if services are to improve, to know whether features recommended in guidance documents are currently offered by services; and whether what a service thinks they offer is actually experienced by young people.

### Aims

For a cohort of young people with long term conditions in transition between child and adult services:

- To describe the extent to which service providers offer nine proposed beneficial features of transition services
- To describe the extent to which young people report they experienced these proposed features
- To compare the reports of young people with what service providers stated they offer and to investigate reasons for differences

Regarding condition specific comparisons, we expected that young people attending diabetes services would be more likely to have experienced the proposed beneficial features compared to young people with cerebral palsy (CP) or young people with autism spectrum disorder (ASD) and additional mental health problems, because services for those with type 1 diabetes are considered to be more developed [6].

## Methods

### Participants

Three hundred and seventy-four young people aged 14 years to 18 years 11 months using child healthcare services in a range of UK locations were recruited to the longitudinal study of healthcare transition, starting in summer 2012. All the young people with type 1 diabetes or ASD with additional mental health problems were approached in children’s services in five and four UK healthcare provider Trusts respectively. Young people with CP were approached from two regional population registers, the North of England Collaborative Cerebral Palsy Survey and the Northern Ireland Cerebral Palsy Register and there were eighteen healthcare provider Trusts from which participants with CP came. 150 young people with type 1 diabetes, 118 with ASD and 106 with CP joined the study. Those recruited had no significant learning disability, as assessed by the referring healthcare professional, and all could self-report. A parent or carer for each young person was also invited to take part in the study. At a first home visit, made by research assistants to all the young people, baseline data were collected. The wider longitudinal study involved three subsequent visits to all young people who remained in the study, one year apart (see open access published protocol [10]). For the purposes of this report, we use data collected at the first follow up visit and this aligns with the information we had about the features each service said it offered (see below).

Thirteen young people, purposively sampled from the longitudinal cohort, took part in a qualitative sub-study. They were sampled on the basis of age, gender, medical condition, geographical location and stage of transition. A further 34 of their parents, healthcare professionals or significant others were involved in interviews or observations of consultations. The purpose of this qualitative work was to understand better how PBFs were experienced and how young people related them to their overall transition experience. Data collection for this subgroup began during the first year of the longitudinal study and some data collection did not complete until the third year of the study (see published protocol [10]).

### Measures

1) Experience of proposed beneficial features (PBFs) as reported by the young person.

A questionnaire (see Additional file 1) about the features of services experienced by the young person over the preceding twelve months was completed at home visits by research assistants in discussion with the young person (often also with the parent). This was supported by information recorded by the young person about their clinical appointments during the year. In addition, research assistants consulted the medical records of each young person to extract information about provision of any of the PBFs. This included whether the clinic had a staff member with the role of transition co-ordinator (which was not directly asked to young people). Each PBF was recorded as having been received or not.

The definitions of the nine PBFs are presented in Table 1.

**Table 1:** Definitions of the nine proposed beneficial features

Age-banded clinic. An intermediate clinic setting such as a young person’s clinic or a young adult team. In child health services, it would mean that children less than about 14 years would not be at the clinic. In adult services, it would mean adults over 24 years of age would not be at the clinic.	
Meet adult team before transfer. This could be in a joint clinic where child health and adult healthcare professionals consult together; or an adult clinician might visit the child clinic to be introduced; or the young person might have been taken to the adult clinic by their key worker or child healthcare professional to meet the adult clinician(s).	
Promotion of health self-efficacy. The young person is asked ‘Have you received enough help to increase your confidence in managing your condition?’ To fulfil this PBF, the clinic should have a written policy about how they provide information and encourage the young person to take responsibility for their health and give them information about their condition.	
Written transition plan. This should be created some time before transfer. It should include plans for wider aspects of transition, not just the arrangements for transfer to adult health services. The young person should have a copy of it and it should be reviewed at each appointment and updated as necessary.	
Appropriate parent involvement in their child’s care, but with changing responsibilities. Parent and young person are asked separately if they think the level of involvement is appropriate. Involvement concerns what happens in the clinic (parent being present or not and who does the talking).	
Key worker. This is a single person known to the young person whom they can easily contact or go to if there were any problems of co-ordination or misunderstandings that needed to be sorted out. The role could cross into education and social services. Whilst a clinic may have a policy to ‘appoint’ a key worker, this needs to be negotiated with the young person who may report it to be someone else they feel most comfortable with.	
Coordinated team. Some young people need to see a team of people; for example, those with diabetes may need to see doctor, nurse, dietician, and psychologist. Those with cerebral palsy may need to see doctor, physiotherapist, and orthopaedic surgeon. The members of these teams need to work and communicate well together, and demonstrate to the young person and family that this is happening. Coordination of appointments on the same day is one demonstration of such coordination.	
Holistic life-skills training about education, gaining employment, finances, housing, social relationships, sexual health, substance misuse, mental health etc. as well as health maintenance. The young person is asked whether they have had any formal life-skill training offered relevant to their long term condition. The health service may not provide such training but during consultations staff should inquire about such matters and make referrals to other agencies as needed.	
Transition manager for clinical team. This person may not be known to the young person, but should facilitate good working relationships between adult and child services; ensure appropriate materials are available (such as health education or the transition plan); and will monitor that the young person has a suitable appointment in adult services and whether the appointment is kept.	

Where a young person was seen by only one member of a service, the feature *coordinated team* was recorded as ‘not applicable’.

2) Provision of PBFs as reported by the clinical service.

Each service attended by the young people in our cohort was asked to complete a questionnaire about the model of transitional care they provided. The questionnaire (see Additional file 2) mainly focussed on the nine PBFs. The reporting clinician was not asked about the care provided to individual young people.

3) Background data.

Socio-demographic data (date of birth, gender) were captured at the baseline home visit. At the second visit the date of the final appointment (the ‘transfer date’) in child services was recorded if the young person had transferred to adult services or had been discharged to local community primary care (general practitioner care) by the time of the second visit.

### Qualitative methods

Qualitative interviews were undertaken with young people. Interviews with family members and health professionals, along with observations of clinical consultations, were also conducted to gain further insight into how proposed beneficial features were experienced and understood. Where possible, after a minimum of one year, a second interview was undertaken with young people, family members and health professionals. Following the second interview, if the young person had transferred from children’s services, an adult health provider was approached. Interview schedules developed over time. Initially, they were informed by the team’s experience and the relevant literature, and then iteratively refined following analyses. They focused on the young person, in a holistic sense, including health condition, alongside transition and transition services. Follow-up interviews also focused on any change over time, as well as exploring analytic questions from prior analysis across the data set.

All interviews were audio-recorded, transcribed and edited to ensure anonymity. Contemporaneous field notes were made about the non-participant observations in clinical settings.

### Analysis

We undertook the analysis of each data set independently and then compared and integrated them. We undertook the following pre-specified analyses to address study aims:

1. Characteristics of young people successfully followed up after twelve months were compared to those who withdrew from the study in terms of gender, age, condition and site to assess potential bias, using chi-squared or t-tests as appropriate.
2. PBFs experienced by young people are presented descriptively by condition. For *meeting a member of the adult team*, which is time-sensitive, consideration of the timing of an individuals’ transfer from child services was included. The PBFs reported by the service providers are presented descriptively and compared with the reported experience of the young people who attended each service.
3. Analysis of transcripts and field notes followed the standard procedures of qualitative analysis [12]: systematic coding, searching for, reviewing and refining themes [13], first-generation grounded theory - constant comparison, memoing [14], as well as case summaries [15]. Independent coding and cross checking was undertaken, and some data were analysed collectively where the research team shared and exchanged interpretations of key issues emerging from the data. Illustrative findings are presented in relation to issues suggested by the quantitative analysis of the PBFs, for example, to understand discrepancies in accounts between young person and service provider.

## Results

### Comparison of young people followed up with those lost to follow-up

Data were collected at follow-up at twelve months from 304/374 (81%) young people (128 with type 1 diabetes, 91 with ASD and additional mental health problems, 85 with CP). Seventy young people were lost to follow up. Thirty of these could no longer be contacted. Of those who said they did not want to continue, the main reason was no longer being interested but most did not give a reason. There were no significant differences between the seventy young people lost to follow-up and those retained in the study by gender (*p* = 0.28), age (*p* = 0.90), condition (*p* = 0.21) or recruitment site (*p* = 0.20 for diabetes sites, *p* = 0.19 for cerebral palsy sites and *p* = 0.52 for autism sites).

### PBFs reported by the young person as having been experienced during their clinical care over the previous year

Table 2 summarises how many young people received each PBF, as reported by young people and/or extracted from medical records. *Written transition plan* and *meeting adult team before transfer* stand out as rarely experienced. Even when the latter was restricted to young people who had just transferred (before or within two months of the date of their twelve-month follow up research visit), only 31% had experienced this PBF.

**Table 2:** Young people’s experience of proposed beneficial features, overall and by condition

	All conditions *N* = 304	ASD *N* = 91	CP *N* = 85	Diabetes *N* = 128
Proposed beneficial feature	n (% of N)	n (% of N)	n (% of N)	n (% of N)
Age-banded clinic	122 (40%)	5 (5%)	11 (13%)	106 (83%)
Meet adult team before transfer	54 (17%)	4 (4%)	11 (13%)	39 (30%)
Promotion of health self-efficacy	216 (69%)	53 (58%)	41 (48%)	122 (95%)
Written transition plan	41 (13%)	3 (3%)	5 (6%)	33 (26%)
Appropriate parent involvement				
*Young person happy with it*	222 (73%)	66 (73%)	59 (69%)	97 (76%)
*Parent happy with it*	216 (71%)	67 (74%)	52 (61%)	97 (76%)
*Young person or parent happy with it*	265 (87%)	82 (90%)	69 (81%)	114 (89%)
*Young person and parent happy with it*	173 (57%)	51 (56%)	42 (49%)	80 (62%)
Key worker	132 (43%)	35 (38%)	13 (15%)	84 (65%)
Coordinated team Not applicable	170 (56%) *52* (17%)	25 (27%) *37* (41%)	31 (35%) *11* (13%)	114 (89%) *4* (3%)
Holistic life-skills training	101 (33%)	17 (19%)	12 (14%)	72 (56%)
Transition manager for clinical team	45 (15%)	14 (15%)	9 (11%)	22 (17%)

*ASD* autism spectrum disorder with additional mental health problems, *CP* cerebral palsy

As expected, smaller percentages of young people with CP and ASD reported receiving most of the PBFs, as compared to young people attending diabetes services. There were two exceptions to this pattern. First, similar percentages of young people and/or parents across the three conditions were happy with the level of *parent involvement* (56, 49, 62%); second, similar percentages had a *transition manager for clinical team* (15, 11, 17%).

### PBFs reported by the service as provided

There were seventeen providers of services for those with CP and five for those with diabetes. Those with ASD came from four provider Trusts but, due to procedures varying between clinical teams, there were twelve responses in all.

**Fig. 1:**
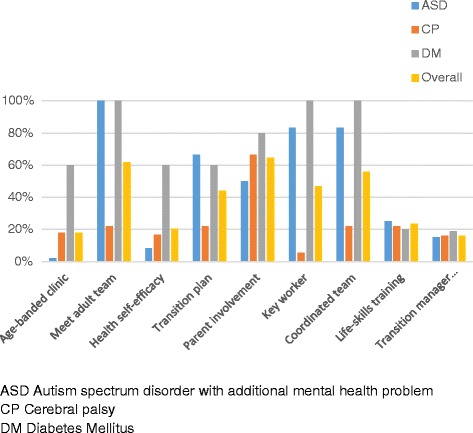
Percentage of services which offer each proposed beneficial feature, by condition and overall

The PBFs which the service stated were provided are shown in Fig. 1. The largest proportions of services reported that they provided opportunities for young people to m*eet adult team before transfer,* had an allocated *key worker,* encouraged *appropriate parent involvement* and provided a *coordinated team.* Few services had a designated *transition manager for clinical team, holistic life-skills training* or *age-banded clinic.* For most of the features, services for those with diabetes provided considerably more than services for those with ASD, which in turn provided more than services for those with CP.

### Comparison between PBFs experienced by young people and the PBFs that the service reported it provided

The extent to which young people reported that they experienced the PBFs which their service reported it provided, is shown in Table 3. For *meeting the adult team before transfer*, only 19% of young people experienced this service feature when the service stated it was provided. The agreement increased to only 39% in the 82 young people who had just left child services and whose service said it had provided a meeting with the adult team. For *transition manager for clinical team* only 25% of the young people in services stating they provided it had evidence for it in their records.

**Table 3:** Extent of agreement about proposed beneficial features comparing what services reported they provided with what young people experienced

	Number of young people attending services which provide the PBF	Number (%) of young people in previous column who reported experiencing the PBF
Proposed beneficial feature (PBF)	N	N (%)
Age-banded clinic	136	105 (77%)
Meet adult team before transfer	231	44 (19%)
Promotion of health self-efficacy	86	69 (80%)
Written transition plan	108	32 (30%)
Appropriate parent involvement	208	
*Young person happy with it*		160 (77%)
*Parent happy with it*		155 (74%)
*Young person or parent happy with it*		188 (90%)
*Young person and parent happy with it*		127 (61%)
Key worker	192	95 (49%)
Coordinated team	195	134 (69%)
Holistic life-skills training	62	27 (43%)
Transition manager for clinical team	58	15 (25%)

There was somewhat better agreement for three more PBFs: *written transition plan*, *holistic life-skills training* and *key worker*, where 30, 43 and 49% respectively of young people experienced the feature when the service stated it was provided.

The agreement between the service statement and young person’s experience of *appropriate parent involvement* was high (77% young people and 74% of parents/carers happy with the level of parent involvement). For the young person and their parent/carer to both be happy with level of involvement gave a lower percentage of 61%. Agreement about attending an *age-banded clinic* was high (77%) as was *promotion of health self-efficacy* (80%). C*oordinated team* also had reasonable agreement (69%) between young people’s experience (where this was applicable) and what the service stated was provided.

There were two PBFs where the young person said they experienced the PBF but the service said it did not provide it (data not shown). Having a *key worker* was reported as experienced by 31% of young people where the service stated that a *key worker* was not provided. *Promotion of health self-efficacy* was reported as experienced by 65% where the service stated it was not provided.

### Variation in meaning of PBFs as experienced by young people, families and service providers – The qualitative interviews and observations

#### Age-banded clinics

Of the young people in the qualitative study, only those with diabetes reported attending an *age-banded clinic*. We observed the appointments of two young people (Ruth and Jack) with diabetes attending adult outpatient clinics designated as age-banded. However, the patients in the waiting area included adults much older than 24 years of age. Neither of the young people reported this experience as problematic; one indicated that she was ‘not really fussed by that’ (Ruth). However, both their parents and healthcare professionals discussed a range of concerns about their experience attending the clinic. The mother of Jack, described it as ‘quite frightening I guess is the word’, and Ralph (father of Ruth) described it as ‘not intimidating but’ he thought that the young people would be ‘more comfortable’ without seeing such older people.

It is a young adults' clinic, but you go and there's no other young people there. Should they stagger them? You're sitting with people in wheelchairs, with amputees, the great, big older people that have obviously got type two (diabetes). It's not a young clinic unless you sit there and everyone's between 18 and 25 (Judy, mother of Jack)

The diabetes healthcare professionals raised similar points.

I think what we know is that it can be very off-putting for young adults, say, for example, you know, if the waiting room is full of people with a very different demographic, you know, elderly, amputees or blind people, or whatever. Not that they shouldn’t see those people, but I think, you know, it’s partly it plays on their fears for what the future holds (Dr Redgrave, diabetes consultant paediatric physician)

Parents and healthcare professionals thought that young people needed protection from issues they may need to face as adults with a long term health condition, while at the same time saying that reality should not be hidden from them.

All thirteen young people interviewed reported a preference for appointments in outpatient clinics set-up specifically to support young people (rather than in child services with play activities for babies and younger children). The young people preferred to be in environments with other young people. However, they valued the quality of the interactions with their healthcare professionals above the physical and visual space of the waiting area. They wanted to see healthcare professionals they can ‘trust’, that are ‘nice … helpful if I ask any advice’ (Ruth) and with whom they can build ‘rapport’.

*Age-banded clinics* were considered useful by healthcare professionals. Such clinics appear to focus the minds of healthcare professionals, from both children’s and adults’ services, on their interactions with young people and assist their provision of developmentally appropriate healthcare.

I think they work well and I think the reason that they work well is that it focuses your mind on adolescent issues. I don’t know whether it works well for the patients but it gets you into a mind-set of thinking about those sorts of things. Whereas, if you’re going from 18 months old to an 18 year old, you’re sometimes not in the right zone (Dr Ardo, diabetes consultant paediatric physician)

#### Promotion of health self-efficacy

Some young people and parents described receiving support in developing the young person’s health self-efficacy, but that this ‘was never sort of formal’ (Penelope). This work seemed to occur at different times in consultations with a range of healthcare professionals, as well as beyond the service in organised trips and weekends away. For some, ‘promotion of health self-efficacy’, albeit through informal means, was ‘all the way through’ (Amy, mother of Angela) their experience of the clinical service.

Many healthcare professionals offered a similar narrative, where such work was considered to be an ongoing part of consultations ‘always a side-line of the work anyway’ (Ms White, speech and language therapist). This was not based on a specific policy, but rather ‘a gradual process’ (Dr Ardo, diabetes consultant paediatric physician); only one service described a more formal process to ensure consistency across the team. Primarily, this aspect of clinical care was reported to occur over time and was not seen as tied to specific educational events.

#### Written transition plans

In an already bureaucratic culture, some professionals were sceptical about the place of such documents. One practitioner noted ‘I’d like to see patients and facilitate effective transitions, not be filling in plans’ (Dr Peters, consultant psychiatrist). Another said:

I think unless it’s been, sort of, discussed with the young person it’s, sort of like, completely hopeless. … It’s, it’s one of those things that people look at when it’s written and then never again. And actually does anyone pay any attention to it? I don’t think health professionals do. And I don’t think the young people do (Dr Redgrave, diabetes consultant paediatric physician)

The practicality of fitting into consultations discussion about the *transition plan* and its completion was also seen as a barrier. One service had implemented *written transition plans* as part of a research project, but had discontinued them when the project ended. Two services viewed such plans as an ‘aspiration’ or ‘target’. For most healthcare professionals, plans around transition were seen as occurring over a series of consultations which were then recorded in clinic letters.

[They are] laid out in the kind of letters which I write to the young person and their parent … And transition is part of that, we don’t have a separate piece of paper that’s called transition, it’s everything weaved into their clinic letters (Dr Carlos, rehabilitation physician)

Some young people reported experiencing plans about transition over time – ‘It was more just something we talked about, there wasn’t really anything written down’ (Darryl). None of the young people was aware of being offered any sort of ‘written transition plan’. One young person reported having ‘got loads of transition plans’ (Fran) centred on her move from school to college, but nothing from her health services. In another case, a written plan was reported but only the young person’s mother was aware of it. The mother (Jane, mother of Jonah) said ‘It’s here somewhere’ and then pulled a document called a ‘transition planner’ from under a pile of documents. Angela’s mother noted:

Well, we have nothing at all, to the extent that I don’t even know when she goes over. I’m assuming it’s eighteen but I don’t know (Amy, mother of Angela)

Another parent noted that ‘anything would have been useful’ (Beryl, mother of Ben). In such a vacuum, parents and relatives stressed the potential value of a written plan for young people, as well as for themselves. For Paul, brother of Penelope, ‘it seems pretty obvious really, like, that you would need something like that’. The young people were aware of potential problems, especially around their ability to ‘lose’ such documents, which contained personal information. Parents, relatives and healthcare professionals also shared this concern that health-related paper documents will be lost.

## Discussion

There is much agreement in national and international recommendations about the importance of the proposed beneficial features (PBFs) we studied. The nine PBFs include six recommended in the publication of UK national guidance to health and care providers NICE [4] – *meeting the adult team, promotion of health self-efficacy, written transition plan, appropriate parent involvement, key worker, holistic life-skills training.* Nevertheless, our study shows that implementation in 2013/14 was patchy both in terms of what services for young people say they provide, and what was experienced by young people themselves. Fewer than half of services (Fig. 1) stated that they provided *age-banded clinic, individualised written transition plans, transition manager for clinical team, protocol for promoting health self-efficacy,* or *holistic life-skills training*.

Services for young people with diabetes were more organised for supporting healthcare transition than services for young people with CP or ASD with additional mental health problems. Nevertheless, only 26% of young people with diabetes (Table 2) reported having a *written transition plan*, and only 30% of those who had already had their final appointment in child services had *met a member of the adult diabetes team before transfer*. Therefore, there is much room for improvement across all the services that the young people recruited to this UK–wide study were attending.

Why might diabetes services be better organised than those for young people with complex disability? First, diabetes services have had an interest in improving services for transition for at least a decade, with many resources proposed to assist the process [6]. Second, haemoglobin A1c levels peak between ages 14 and 18 before gradually declining [16]. Therefore, this age period is likely to have been a target for careful diabetes management and improved services. Third, unlike in diabetes, it is often unclear to where to transfer the healthcare of young people with ASD [17] or CP [18], and there may be very different (usually more restricted) criteria for accessing adult health and mental health services.

*Age-banded clinics* were reported as a frequent feature (60% Fig. 1) of the services provided for those with long term conditions such as diabetes. Such provision can be relatively easily documented within the service specification and clinical profiles. However, the qualitative data found that implementation was less straightforward and that the intention of the healthcare professionals, to be primed to think about adolescent or young adult needs, may not extend to the family’s experience especially in the waiting area. The young people interviewed and the UP group, commented that *age-banded clinics* per se may not be crucially important. What is important is whether or not the young person feels comfortable in talking with the healthcare provider. A recent systematic review of twenty two studies of youth-friendly healthcare drew a similar conclusion – that staff attitudes are universally reported as important, whereas other aspects, such as an appropriate environment, are specific to particular contexts [19].

A second feature that should be able to be clearly planned and documented by a service is some kind of *written transition plan*, even though the format may vary from individual to individual. Over all conditions, this was the least frequently experienced PBF (13% Table 2), particularly for the young people with disabilities (CP and ASD with additional mental health problems). There was considerable resistance amongst healthcare professionals to *written transition plans,* yet without such documentation the young person and family can be left ‘in the dark’ about what will happen after healthcare transfer to adult services. Some young people and parents also said there were practical difficulties in maintaining a written document and remembering where it was kept. The UP group commented that different types of formal paperwork can be helpful (such as a ‘health passport’) but only where it is clearly valued by the healthcare professionals by being read and updated during consultations. Adoption of a tested format (such as ‘Ready, Steady, Go, Hello’ [20] which is endorsed by NHS England) http://www.uhs.nhs.uk/OurServices/Childhealth/TransitiontoadultcareReadySteadyGo/ForhealthprofessionalsReadySteadyGoresources.aspx, could be made more widely available, including in electronic formats, and might be useful as a way to operationalise the NICE transition guideline [4] on creating a ‘personal folder’ for the young person to share with adult services.

Another issue is how to enable the role of a *key worker* (or ‘named person’ in NICE guidance) within the transition procedures. This is a recurring theme for those working in disability services. The introduction of the role of *key worker* has been repeatedly recommended in policy documents from the Court Report [21] onwards [22]. Implementation has been patchy and difficult in any formal structured way; yet many individuals and families will identify a person whom they trust and can rely on to answer questions and sort out difficulties. In the current study, a third of young people reported having a key worker when the service said this was not a feature they provided.

For young people, longer term, trust-based relationships with specific healthcare professionals seemed key to enabling them to develop confidence in making sense of and learning how to manage their condition. Thus, *promotion of health self-efficacy* was experienced as an integral part of the interaction during consultations, rather than as a specific event. This gradual model of delivery is a strength where it works well, but may therefore be dependent on clinician style and difficult to document. Issues of timing and pace of transition are important [23] and need to be individualised.

PBFs do not necessarily stand alone. For example, in a service which has the feature of *transition manager for clinical team*, this might in turn mean it is more likely that there is systematic logging of who the *key worker* is and ensuring that *written transition plans* are implemented. A quality-improvement programme (of PBFs) has been shown to make a difference. Dutch healthcare teams [24] were trained to use a toolkit of strategies, which included creating a transition protocol, having a transition coordinator, and promoting individual self-management plans with young people. They found that young people’s satisfaction with transitional care was related, around twelve months later, to better emotional health and quality of life.

### Strengths and limitations of the study

The strengths of the study lie in the spread of exemplar conditions included, the large number of young people recruited and retained, the completeness of response from the services attended and the use of mixed methods. Furthermore, it is the first study since publication of the NICE Transition guideline [4] to report on the extent to which proposed elements of good practice are actually provided by services for young people with long term conditions.

One hundred four (34%) young people had transferred by the second visit. However, the purpose of our analysis was not to compare pre and post transfer. The proposed beneficial features are relevant to adult and child services. Transition is a process occurring over many years and intended provision and actual experience of features is relevant throughout transition.

Our study did not include the model of transitional care provided by adults’ services; any further study should do so. An international Delphi study [25] recently proposed key indicators for a transition programme with a focus on adult services, including the need for a trusting relationship to be built with an adult service provider, and ensuring timely attendance after transfer.

The interpretation of exactly what each beneficial feature means may vary. Some PBFs were operationalised for service providers in a slightly different manner from the way in which they were discussed with young people. Further, although the research assistants were carefully trained together on interpretation of the PBFs (including what to look for in medical records and regular conference calls to enable consistency to be maintained), the recorded frequencies of PBFs experienced should be interpreted accordingly.

## Conclusion

Observational studies or trials which examine the influence of features of transition services on outcomes should ensure that the experiences of young people and families of the proposed beneficial features are captured. They should not rely on the service specification.

In further stages of our longitudinal study [10] we will capture data about the PBFs again. This will help to determine whether the features are experienced more or less frequently as the young people with long term conditions reach the point of transfer from child services, or following transfer. We shall also examine whether exposure to specific PBFs is associated with better outcomes.

## Additional files

Additional file 1: Beneficial Features Data Summary for Previous Year. (PDF 229 kb)

Additional file 2: Clinic/service models. (PDF 180 kb)

## Abbreviations

ASD: Autism Spectrum Disorder
CP: Cerebral Palsy
NICE: National Institute for Health and Care Excellence
PBF: Proposed Beneficial Feature
