# Supplementary material for: How well do services for young people with long term conditions deliver features proposed to improve transition?
Source: BMC Health Serv Res. 2018 May 8;18:337. doi: 10.1186/s12913-018-3168-9 (PMC5941647; doi:10.1186/s12913-018-3168-9)
Supplement: Supplementary file 1 — Beneficial Features Data Summary for Previous Year. (PDF 229 kb) [file 12913_2018_3168_MOESM1_ESM.pdf]

Participant ID	
Date and Visit	
Research Associate	

Additional file 1

File name: Colver April 2018 Supplementary Material 1.pdf

Title: Beneficial Features Data Summary for Previous Year

Description of data: Questionnaire about exposure to proposed beneficial features.

Questions to be completed by researcher and young person together

Transition clinic	Have you attended a clinic that was just for young people/adolescents, or young adults, or a joint clinic with staff from both child and adult services?		
Yes		No	
If yes, circle any that apply: Adolescent Joint Young Adult			
Adult team	Did you meet the adult team or anyone from the adult team while planning to move, or before you moved, to adult services?		
Yes		No	
If yes, circle any that apply: Joint Met for introduction			
Self-efficacy	Have you received enough help from the health services to increase your confidence in managing your condition?		
Enough help	Not enough help	Did not need help	
Plan	Do you have/have you had a written transition plan that talks about your health services?		
Yes		No	
TP may be held in the medical notes; possibly separate page with heading, or checklist/questionnaire. If YP has a plan created in education, check it also includes mention of health services.			
Parent involvement (a)	Has your parent/carer been involved and consulted in your healthcare transition?		
Yes		No	
(b)	Was it at the right level?	Was it at the right level?	
Yes	No, too little	No, too much	Yes No, too little
Key worker	Have you got or had a key worker who works with you? i.e. a single person you approach to sort out any problems around your health care?		
Yes		No	
	If yes, note who it is, and profession:		
The KW can be from another service, but it is recognised that they can influence health provision. If the YP does not know the profession, please find out.			
Coordination	Can you see a variety of professionals (such as doctor, psychologist, therapist or dietician) on the same day or place?		
Yes	No	No – only in contact with one professional	
	Add comments		
The point is whether they seem to work as a team, so note any comment about 'they don't seem to			

Please turn over →

communicate with each other', etc.		
Life-skills training	Have you received formal training (such as going on a course or to a meeting or watching videos etc.) in thinking about and planning your future - that is, relationships, education, training, work, finances, etc.? This would be training related to having your condition.	
Yes		No
The training would be holistic, not just about managing the YP's health condition.		

Question for parent/carer:

P (c)	Do you feel you have been involved and consulted about your young person's health?			
Yes			No	
P (d)	Was it at the right level?		Was it at the right level?	
Yes	No, too little	No, too much	Yes	No, too little

Question from medical records:

Co-ordinator	Is there evidence of a coordinator who makes sure services for transition work well together?	
Yes		No
