# Supplementary material for: How well do services for young people with long term conditions deliver features proposed to improve transition?
Source: BMC Health Serv Res. 2018 May 8;18:337. doi: 10.1186/s12913-018-3168-9 (PMC5941647; doi:10.1186/s12913-018-3168-9)
Supplement: Supplementary file 2 — Clinic/service models. (PDF 180 kb) [file 12913_2018_3168_MOESM2_ESM.pdf]

Additional file 2
File name: Colver April 2018 Supplementary Material 2.pdf
Title: CLINIC/SERVICE MODELS
Description of data: Questionnaire about transition service provided at each site.

CLINIC/SERVICE MODELS
Transition Longitudinal Study

As part of the Transition Programme longitudinal study, we need to document the service models each year so that we can identify changes during the course of the study.

Also it may be useful to know if there are major discrepancies between how the patient and the service perceives the service.

We ask you to provide information which reflects your current practice **over the last year** (i.e. 2012/2013) rather than proposed service developments.

The questions are about the clinics in which young people eligible to take part in the Transition Longitudinal Study have been seen in the last year. That is, young people with
(1) diabetes, or
(2) cerebral palsy, or
(3) autism spectrum disorder (ASD) with additional mental health difficulties (and with average range intellectual ability or at most mild learning disability).

At recruitment the young people were aged 14 years to 18 years 11 months and pre-transfer to their adult healthcare.

We expect that one person will complete the questionnaire, but we think it would be helpful to discuss the answers within your service/clinic team.

Allan Colver and Helen McConachie

NB: Please answer with respect to a single site or service team.

**Centre, Hospital Site or Service name, and Trust name**	
**Clinic name**	
**Transition grouping:**<br>**1. Diabetes**<br>**2. Cerebral Palsy**<br>**3. ASD with mental health problems**	
**Date of completion of questionnaire**	
**Name of lead Consultant(s)**	
**Name of person completing the form (if different from above)**	

CURRENT SERVICE PROVISION

1. **At what type of clinic has your service seen the young people recruited to the Transition study?**
   Please tick and give age criteria. Please put a cross against any type you provide

Clinics

**Clinic type:**	**Age criteria for the clinic**
Children and young people (all ages)	
Dedicated adolescent (defined as 11+ yrs)	
Joint transition clinic (shared staff with adult service	

TRANSITION COORDINATION

3. Is there a person (who may or may not be an actual clinical team member) who has a specific transition coordinating role for the clinic or service (i.e. not a key worker for individual young people)?

1. Paediatric team member………………………………………………….…… **YES❑/NO❑**
2. Adult team member ……………………………………………………….…..**YES❑/NO❑**

If YES, is this defined in their job description and job plan? **YES❑/NO❑**

CURRENT TRANSITION MODEL

What is the PREDOMINANT current transition model in your service? *(Choose one option only; this includes the option for mixed provision under **Other**.)*

(i) Single doctor? .... **YES** ❑

(Dually trained consultant. Patient moves from paediatric to adult clinic but remains under same consultant throughout)

If **YES**, does the whole team remain the same or are there changes in nurse, psychologist, occupational therapist, etc? …………… … …… ……… **YES** ❑

Please give details…………………………………………………………………………………

……………………………………………………………………………………………………

(ii) Combined clinic? . **YES** ❑

(Different paediatric and adult teams but includes combined clinic at one site from which young people are moved into the adult service)

What is the average duration the young person remains in combined clinic prior to transfer to adult service?..

(iii) Paediatric to adult teams – direct transfer **YES** ❑

(Different teams, no overlap)

(iv) Age banded clinics throughout – a 'developmental model' **YES** ❑

(e.g. 0-11 to adolescent clinic (11+) to adult service (18=) +/- an interim young adult clinic (16-25) in the adult setting)

(v) No adult service (i.e. responsibility reverts to young person and GP) **YES** ❑

(vi) Other **YES** ❑

(Please give details including if it is a combination of some of the above)

……………………………………………………………………………………………………

……………………………………………………………………………………………………

……………………………………………………………………………………………………

……………………………………………………………………………………………………

SPECIFIC ASPECTS OF SERVICE

	YES	NO	Comments	Not applicable
Young people have an opportunity **to meet members of the adult team** before transfer of care				
If YES				
In joint clinic				
Or<br>Meeting outside a joint clinic				
Staff discuss with young people and parents the level at which they wish **parents** to be involved in consultations				
Individualized **transition plans** are routinely used for all patients in the service.				
The service has a protocol for promoting **health self-efficacy** (i.e. actively helping young people to feel confident in managing their health condition).				
The service ensures that each young person has **a key worker**, i.e. a single person they approach to sort out any problems around their health care (may not be formally allocated, and may not be a health provider but in close touch with health services)				
Young people can see a variety of professionals (such as doctor, psychologist, therapist, dietician, where appropriate) on the same day or place (i.e. **a coordinated team)**				
The service provides formal training in thinking about and planning for the future (i.e. relationships, education, training, work, finances, etc.), or checks that the young person has opportunity to access such training **(Holistic life-skills training**).This is training related top having their condition, but not just managing their condition.				

ND questions about age banded clinics and transition manager for clinical team are on Page 2

ADULT SERVICES

Please provide details of the adult clinic/service you most frequently refer young people on to.

Clinic service or name?..

Name (s) of consultant(s)……………………………………………………………………………..

Hospital or setting………………………………………………………………………………………

NHS Trust…………………………………………………………………………………………...

Clinic service or name?..

Name (s) of consultant(s)……………………………………………………………………………..

Hospital or setting………………………………………………………………………………………

NHS Trust…………………………………………………………………………………………...

PLEASE ADD ANY ADDITIONAL COMMENTS

Thank you very much for your time and cooperation.

Please return this survey to:
XXXXXXXXXXX
Transition
Newcastle University, Institute of Health and Society
Sir James Spence Institute 3rd floor
Royal Victoria Infirmary, Queen Victoria Road,
Newcastle upon Tyne NE1 4LP
